# Detection of *Leptospira* in urine of apparently healthy dogs by quantitative polymerase chain reaction in Haryana, India

**DOI:** 10.14202/vetworld.2020.2411-2415

**Published:** 2020-11-12

**Authors:** Preeti Rohilla, Rajesh Khurana, Aman Kumar, Kanisht Batra, Renu Gupta

**Affiliations:** 1 Department of Veterinary Public Health and Epidemiology, Lala Lajpat Rai University of Veterinary and Animal Sciences, Hisar, Haryana, India; 2 Department of Animal Biotechnology, Lala Lajpat Rai University of Veterinary and Animal Sciences, Hisar, Haryana, India

**Keywords:** *Leptospira*, leptospirosis, qPCR, *lipL32*, Haryana

## Abstract

**Background and Aim:**

Leptospirosis is a zoonotic disease caused by bacteria of the genus *Leptospira*. The organism can spread through the urine of infected animals, which can get into water or soil and can survive there for weeks to months. The study was undertaken to detect the pathogenic *Leptospira* in healthy dogs’ urinary shedding by real-time polymerase chain reaction (qPCR).

**Materials and Methods:**

Leptospirosis is a zoonotic disease caused by bacteria of the genus *Leptospira*. To detect the pathogenic *Leptospira* organisms in dogs’ urinary shedding, 239 urine samples were collected from healthy dogs from April 2018 to March 2019 from different areas of Haryana. All the urine samples were processed for DNA extraction and qPCR technique was used to detect the presence of *Leptospira*.

**Results:**

Out of 239 urine samples of dogs, none of the samples resulted in the detection of DNA of pathogenic *Leptospira* organisms.

**Conclusion:**

The present study indicated low risk of transmission of *Leptospira* organisms from dogs’ urine to human beings in Haryana.

## Introduction

Leptospirosis is an emerging and anthropozoonotic disease that mostly occurs in an acute form. It is globally found in all mammalian species [1,2]. The exposure to infection of leptospirosis is prevalent worldwide and the disease is endemic in some tropical and subtropical regions [3]. Dogs, rats, cattle, and buffaloes are the maintenance hosts for leptospirosis. In chronic form of the disease, pathogenic *Leptospira* colonize the renal tubules of maintenance hosts. It is an occupational disease; humans accidentally come in contact with carrier animals or environment contaminated with leptospires [4,5].

At present, dogs are the popular pets transmitting the infection and are the important carriers with a risk of leptospirosis transmission [5]. *Canicola* and *icterohemorrhagiae* are the common serovars causing leptospirosis in dogs. Transmission of leptospires usually occurs between healthy dogs and asymptomatic carriers by the territorial behavior of urine marking and the urine smell of other dogs. Transmission of *Canicola* serovar of *Leptospira* occurs mainly in this way. Fever, vomiting, diarrhea, myalgia, nose bleeding, and jaundice are the common clinical signs of leptospirosis in dogs [4]. The clinical signs progress more rapidly in acute cases of the disease, including hypothermia, bleeding, and liver and kidney failure which leads to death in 2-3 h [4]. Vaccination status of the dog, age, serovar virulence, degree of exposure to the disease, and host immune response may change the intensity of the clinical signs [5-7].

Molecular diagnostic techniques are increasingly being used for clinical diagnosis because these are more sensitive as compared to culture methods. These techniques are faster, organisms can be detected directly in specimens and the infections can be confirmed earlier than serological tests [8]. For the molecular diagnosis of infectious diseases of both man and animals, the development of real-time polymerase chain reaction (qPCR) is a boon to the society. Conventional PCR assays have many limitations such as poor precision, low sensitivity, low resolution, and size-based discrimination only, results are not expressed as numbers, ethidium bromide for staining is not very quantitative and post-PCR processing. To overcome these limitations of conventional PCR, qPCR assays are performed. This assay is easier to perform and less time-consuming, shows reduced variability and contamination, facilitates online monitoring, and does not require post-reaction analyses [9,10]. For the diagnosis of *Leptospira* infection, many authors have described the use of several qPCR assays that amplify different target sequences [11-14]. In majority of these studies, role of the *lipL32* gene, which encodes the *Leptospira* subsurface lipoprotein *lipL32*, has been studied [15]. The gene *lipL32* is believed to be a virulence factor for pathogenic species of *Leptospira*. Thus, *lipL32* gene increases the specificity of molecular methods that help in selective detection of the pathogenic *Leptospira* [16,17].

The epidemiology of *Leptospirosis* in the animal host needs to be understood carefully because it helps in assessing, monitoring, and mitigating the risk of disease in human beings. Based on these observations, the present research work was planned to detect the pathogenic *Leptospira* in dogs’ urine samples. This is the first study for the detection of *Leptospira* organisms in healthy canine population in Haryana state, India.

## Materials and Methods

### Ethical approval

The Institutional Animal Ethics Committee suggested that there is no requirement of permission for given work performed in this manuscript. Hence, ethical approval was not required. However, urine samples were collected without any harm to the animals.

### Collection of samples

Thirteen districts of Haryana state, namely: Panchkula (n=30), Karnal (n=17), Sirsa (n=3), Hisar (n=84), Fatehabad (n=3), Rohtak (n=39), Bhiwani (n=8), Jhajjar (n=2), Charkhi Dadri (n=1), Sonipat (n=1), Gurugram (n=14), Palwal (n=6), and Faridabad (n=31) were included in the present study for the collection of a total of 239 urine samples of dogs. The samples were collected during the period between April 2018 to March 2019. Five to 10 mL of urine was collected from each dog through free catch or urinary catheterization method in sterile sample collection vial.

### DNA extraction from urine samples

All the urine samples were processed for DNA extraction within 48 h after collection of samples using the QIAamp DNA mini kit (Qiagen Inc., Valencia, CA) as per the manufacturer’s instruction [18]. All the urine samples were centrifuged at 10,000 rpm (4°C) for 15 min and 200 μL ATL (lysis buffer) buffer was added to the pellet. Then, 20 μL proteinase K was added and mixed using a vortex machine and kept for incubation at 56°C until the pellet was completely lysed. Then, 200 μL buffer AL and 200 μL ethanol (96-100%) were added to the sample. The cocktail prepared was poured into the QIAamp mini spin column (in a 2 ml collection tube) and centrifuged at 8000 rpm for 1 min. Then, 500 μL buffer AW1 and 500 μL buffer AW2 were added successively for washing and elution was taken in 50 μL AE buffer. This elution containing DNA was stored at −20°C for further use.

### Quantitation of DNA samples

The concentration and purity of the DNA isolated from urine samples were measured spectrophotometrically (BIO-RAD, USA) by measuring the wavelength at A260 and A280 and their purity was assessed by taking the 260/280 ratio [19]. The concentration of DNA present in samples was calculated using the formula given below:

DNA concentration (μg DNA/mL) = OD 260 × 50 × dilution factor

### Design of primers and probes for real-time TaqMan assays

qPCR was carried out to amplify the *lipL32* gene of *Leptospira* organisms. The forward and reverse primers used in this study generated PCR product of size 242 bp. The primer pair and probe used in the study were: F= 45F (5′-AAG CAT TACCGC TTG TGG TG-3′); R= 286R (5′-GAA CTCCCA TTT CAG CGA TT-3′); Probe- 189P (NED-5′-AA AGC CAG GAC AAG CGCCG-3′-NFQ) [20].

### Optimization of qPCR assay

The qPCR amplifications were performed in Stratgene MX3005P qPCR System (Agilent technologies, USA) using TaqMan probes. The plasmid DNA of *Leptospira* was taken as positive control and different dilutions were made for optimization of qPCR. Optimal concentrations of primers (100, 300, 500, 600, 700, and 900 nM) and probes (200, 250, and 300 nM) targeting the *lipL32* gene were tested (Applied BioSystems, Thermo Fisher Scientific Inc., Carlsbad, CA, USA). In the thermal cycler, amplification protocol followed was: Preheating at 50°C for 2 min, initial denaturation at 95°C for 10 min, 45 cycles of denaturation at 95°C for 15 s, and annealing and extension at 60°C for 1 min. To create a qPCR standard curve, a setup of qPCR reactions to amplify different amounts of positive control was made. A qPCR standard curve for positive control of *Leptospira* organism was graphically represented as a semi-log regression line plot of Ct value versus log of input nucleic acid. On Y-axis, Ct value, and on X-axis, quantity of different concentrations of positive control was taken.

## Results

To detect the *Leptospira* in urine of dogs, qPCR method was used because it was more sensitive and specific as compared to the conventional PCR method and a very small quantity of target DNA can be determined in real-time.

### DNA extraction and positive samples

Out of 239 urine samples analyzed, none of the samples was found positive for pathogenic *Leptospira*.

### Quantification of DNA samples

The DNA concentration quantified by spectrophotometer ranged from 2 to 95 μg/mL in samples. A standard curve was created before processing of the study samples.

### qPCR optimization

After evaluation of results, it was observed that 250 nM concentration of each forward and reverse primer and 100 nM of probe gave the lowest Ct value. The qPCR assay was optimized using positive control plasmid in an optimized reaction with a final volume of 10 μL using 0.4 μL of each primer, 0.4 μL of the probe, 5 μL of TaqMan® Universal Master Mix II (Thermo Fisher, Scientific Inc, Carlsbad, CA, USA), 0.8 μL of nuclease-free water, and 3 μL of extracted DNA.

### Limit of detection (LOD) and linearity of assays

**Figure-1:**
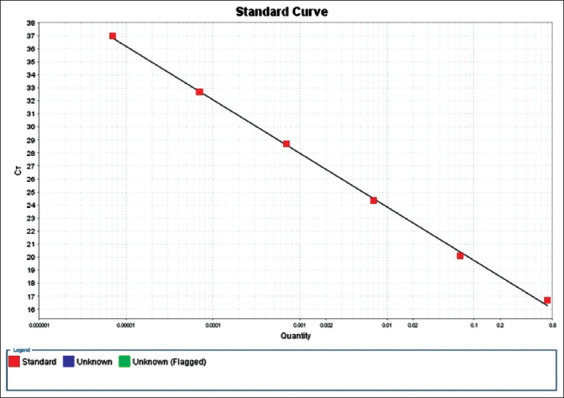
Photograph showing standard curve of real-time-quantitative polymerase chain reaction for detection of pathogenic *lipL32* gene for the detection of *Leptospira*.

**Figure-2:**
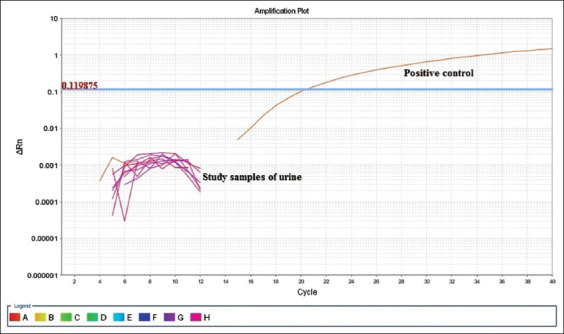
Graph of real-time-quantitative polymerase chain reaction showing the detection of Ct value of only positive control and in study samples Ct undetected in urine samples for diagnosis of canine *Leptospira lipL32* gene.

The standard curve was generated using positive control plasmid having an efficiency of 98.92% with a slope of −3.673 and R^2^ (coefficient of determination) value of 0.995 (Figure-1). The LOD of DNA was 3 ng/mL in sample based on standard curve created for the detection of *lipL32* gene of *Leptospira*. Only positive control was detected by qPCR assay for *Leptospira lipL32* gene with a Ct value of 18.5 (Figure-2). All 239 urine samples showed undetected Ct value for *lipL32* gene of canine *Leptospira*.

## Discussion

In the present study, urine samples were taken from healthy dogs that were not showing symptoms of leptospirosis for the detection of *Leptospira*.

On of basis of previous studies, it has been assumed that clinically normal dogs can be chronic carriers of infection, thus maintenance hosts and shedding *Leptospira* through urine into the environment [21-24]. Hence, urine samples collected from 239 healthy dogs were tested by qPCR. Intensive care was taken that all the extracted leptospiral DNA were preserved at −20°C before processing qPCR assay. However, no sample tested positive, only positive control showed lowest Ct value.

Apparently healthy dogs can transmit leptospirosis in human beings. This seems especially appropriate because there is a consensus that the dogs are more frequently exposed to known risk factors of the disease and thus may act as sentinels of environmental contamination [25-27]. In a study conducted during 2006-2008, 100 blood and 18 urine samples of rodents and dogs were processed for the detection of *Leptospira* organisms by PCR assay. Two blood and five urine samples from rodents were found positive for leptospiral DNA that proves possible role of dogs and rodents in the transmission of leptospires to humans [28]. Similarly, in the present study, all the samples collected from Haryana were also screened retrospectively by qPCR for leptospiral DNA detection but it was not detected in any one of the urine samples.

In a study conducted in Botucatu County, Sao Paulo State, Brazil, urine samples from 106 asymptomatic dogs were collected between October 2014 and June 2015. One (1%) dog was positive by PCR that showed a low prevalence of infection by *Leptospira* spp. [29].

In the region of Algiers, urine samples of 211 stray animals (104 dogs and 107 cats) were collected between April 2017 and November 2017. Out of 107, none of the cat urine samples was found positive, while 5/104 (4.8%) canine urine samples (asymptomatic mixed-breed dogs) were positive in two qPCR assays targeting the *rrs* and *hsp* genes [30]. The efficiency and R^2^ values of generated standard curve for genus *Leptospira* were estimated. The R^2^ value of 0.995 and efficiency value of 98.92% were observed. Similar kinds of results were observed by authors with R^2^ and efficiency values of 0.998 and 98.96%, respectively [31].

The shedding prevalence of *Leptospira* in a study of Germany [32] was 1.5% (3/200), in Switzerland [23] was 0.2% (1/408), and in the USA [21] was 8.2% (41/500). Hence, in the above studies, the detection of pathogenic *Leptospira* organisms by PCR in the urine of clinically healthy dogs justified their role as potential reservoirs and vectors for the canine species. All samples being negative do not mean that the disease was not prevalent in dogs. Some of the possible explanations for not finding any of the urine sample positive for *Leptospira* may be that leptospirosis is a more seasonal disease and flooding and rainy season was not present at the time of sampling. Furthermore, dogs are intermittent shedders and not the regular shedder of the leptospires in their urine. Dogs are renal carriers for *Leptospira* so chances become more to find the organism in their urinary shedding and such type of epidemiological study can be helpful to diagnose the organism in advance to prevent the possible transmission to susceptible in contact human population.

## Conclusion

Based on the results of present study, it can be concluded that urinary shedding of *Leptospira* by healthy dogs is uncommon in Haryana, India. The study showed a low risk of transmission of *Leptospira* organisms from dog’s urine to human beings. However, the influence of intermittent shedding and lack of detection due to preanalytical or analytical factors need to be considered and could potentially lead to a large underestimation of the actual level of renal carriage of pathogenic *Leptospira* in the canine population.

## Authors’ Contributions

PR, RG and RK were responsible for the design of the experimental study. PR, KB and AK performed the experimental work. PR, KB and RK drafted and revised the manuscript. All authors read carefully and approved the final manuscript.
